# Physicochemical and Proteolytic Barriers Limiting Activity of Cpl-1 and Pal Endolysins in Human Circulation

**DOI:** 10.3390/cimb48020231

**Published:** 2026-02-21

**Authors:** Marek Adam Harhala, Katarzyna Gembara, Izabela Rybicka, Zuzanna Maria Kaźmierczak, Paulina Miernikiewicz, Krystyna Dąbrowska

**Affiliations:** 1 Institute of Human Biology and Evolution, Faculty of Biology, Adam Mickiewicz University, 61-712 Poznan, Poland; 2 Laboratory of Phage Molecular Biology, Hirszfeld Institute of Immunology and Experimental Therapy, Polish Academy of Sciences, 53-114 Wroclaw, Poland; katarzyna.gembara@hirszfeld.pl (K.G.); izabela.rybicka@hirszfeld.pl (I.R.); zuzanna.kazmierczak@hirszfeld.pl (Z.M.K.); paulina.miernikiewicz@hirszfeld.pl (P.M.); krystyna.dabrowska@hirszfeld.pl (K.D.); 3 Research & Development Center, Regional Specialist Hospital, 51-124 Wroclaw, Poland; 4 Faculty of Medicine, Wroclaw University of Science and Technology, 50-370 Wroclaw, Poland

**Keywords:** endolysins, bacterial infections, enzybiotics

## Abstract

The growing prevalence of antibiotic-resistant bacterial infections poses a serious burden on healthcare systems worldwide. Endolysins are promising candidates for a new type of antibiotic due to their strong bacteriolytic activity. However, important limitations, including reduced activity and short persistence in the bloodstream, must still be addressed. We evaluated the key physicochemical and biological factors limiting the activity and stability of the endolysins Cpl-1 and Pal in blood. The analysis included ionic composition and strength, pH, bystander proteins, physiological temperature, and proteolytic activity. Our results indicate that the aforementioned factors significantly affect Cpl-1 and Pal, suggesting that physiological conditions in human circulation markedly restrict the anti-bacterial potential of endolysins. To overcome these limitations, we designed a set of Cpl-1 and Pal variants with modified amino acid compositions aimed at increasing their resistance to such physiological constraints. One variant demonstrated improved performance in an ex vivo mouse model and lacked a cleavage site for blood proteases.

## 1. Introduction

The steady rise in antibiotic-resistant bacterial infections has placed immense pressure on global healthcare systems, increasing treatment costs and mortality rates [1,2,3]. This challenge has intensified the search for antibacterial strategies that act through mechanisms distinct from those of traditional antibiotics. Among the most promising candidates are bacteriophage-derived endolysins [3], bacteriolytic enzymes capable of directly degrading the bacterial cell wall. Their extremally high activity makes them promising candidates for combating multidrug-resistant pathogens, including *Streptococcus pneumoniae* [4,5,6].

To date, relatively few clinical trials have investigated endolysins, including engineered variants, for potential market introduction. For example, LMN-201, tested in combination with three toxin-binding proteins (NCT05330182) [7,8,9,10,11,12], represents one such candidate. Some of the tested proteins are chimeric or otherwise modified (NCT02439359, NCT01855048) [7,13,14]. Despite ongoing trials, the 2025 World Health Organization (WHO) report listing antibacterial treatments targeting WHO bacterial priority pathogens, *Clostridioides difficile* and *Helicobacter pylori*, identifies only one endolysin-based agent currently in clinical trials [7]. Various proteobiotic modifications, such as PEGylation, glycosylation, fusion to other proteins (e.g., albumin-binding domains), or epitope depletion, have thus far proven insufficient to overcome the common limitations of therapeutic proteins [15,16]. Although simpler strategies, such as dimerization, may improve selected properties, none have successfully addressed the critical challenges required to develop novel, non-traditional antibacterial treatments capable of maintaining enhanced lytic activity in blood serum, progressing through clinical trials, and ultimately reaching clinical application [17,18]. These observations highlight the need for new strategies to modify existing therapeutic proteins.

Two bacteriophage endolysins active against *Streptococcus* spp., Cpl-1 and Pal, have demonstrated strong antibacterial potential in both in vitro and in vivo models [19,20]. However, as with many other bacteriolytic proteins, their clinical use is limited by several factors, including a marked reduction in activity in complex biological environments such as blood [21]. Previous research has indicated that endolysin activity may be altered by physiological factors, including ionic composition in body fluids. Fluctuations in ionic strength and the presence of divalent cations such as calcium and magnesium can significantly impact endolysin activity [22]. Additionally, the high protein concentration in blood may interfere with enzymatic function by altering structural conformation or through competitive interactions with the bacterial substrate, ultimately reducing efficacy [3,23,24]. Furthermore, blood and other body fluids contain proteases capable of rapidly inactivating protein-based therapeutics [25]. Thermal stability and optimal pH ranges of proteins also vary considerably. Particularly, prokaryotic proteins may exhibit temperature and pH optima that differ substantially from physiological conditions in mammalian hosts during treatment.

Electrostatic interactions between proteins and surrounding molecules in ionic solvents are key factors that may affect endolysin function, yet their specific roles remain poorly understood [26,27,28,29,30,31]. The detrimental effect of ions on bacteriolytic activity has been attributed to interference with interactions between the cell wall-binding domain (CBD) and the bacterial cell wall. Our study addresses this by considering the shielding effect of ions present in high-ionic-strength environments, which may reduce the probability of effective CBD-cell wall interactions required for lysis. Some studies further suggest that Ca^2+^ and Mg^2+^ ions stabilize the bacterial cell-wall, thereby decreasing susceptibility to enzymatic degradation [32,33]. Importantly, ions often serve as cofactors necessary for enzymatic catalysis, but such dependence has not been observed for Pal or Cpl-1 [34,35].

Shielding effects arise when a charged macromolecule, such as a protein, is suspended in a polar solvent, like water, containing dissolved ions (e.g., potassium, magnesium, calcium). Surface-exposed charged amino acids generate an electric field that attracts solvent molecules and oppositely charged ions. These ions accumulate around the protein, effectively shielding it by reducing the strength of its interactions, from electrostatic interactions with other molecules or surfaces in the solution [26,29]. Additionally, human and animal blood contain numerous active proteases, both soluble and cell-associated, that play essential roles in immune defense and protein homeostasis [36,37,38]. These proteases are capable of degrading exogenous proteins, particularly those of prokaryotic origin, as part of the host’s innate immune surveillance. Because such proteins have not evolved under mammalian physiological conditions, including proteolytic environments, they are often highly susceptible to rapid cleavage. Although this degradation serves as a protective host mechanism, it may limit the therapeutic efficacy of protein-based drugs. Previous in vivo studies have demonstrated that degradation of injected peptides begins immediately upon contact with blood proteases, substantially shortening their half-life. Nevertheless, specific observations regarding these effects on endolysins, particularly Cpl-1 and Pal, remain limited [25,39].

Importantly, evaluating and comparing the antibacterial activity of endolysins presents methodological challenges. Although endolysins degrade bacterial peptidoglycan (PG), the rate of PG degradation does not necessarily correlate with therapeutic efficacy, which depends primarily on the rate at which bacteria become metabolically inactive. Conventional assays used to assess antibacterial activity have several limitations, including interference from sample turbidity (which affects blood and serum measurements) and/or the inability to provide real-time detection, as in plating-based viability assays. Therefore, in this study we employed a fluorometric assay previously validated in our laboratory [40,41,42]. Shortly, in this assay, metabolically active *S. pneumoniae* cells are incubated with the bacteriolytic agent (here: endolysin) in the presence of the DNA-binding dye Sytox Green. Once bacterial cell wall disruption renders cells metabolically inactive, intracellular DNA becomes accessible to the dye, resulting in increased fluorescence intensity. This approach enables precise, real-time measurement of bacterial loss of viability under controlled experimental conditions. Accordingly, we used this assay to evaluate the effects of the investigated physiological factors on endolysin activity and stability

**Figure 1:**
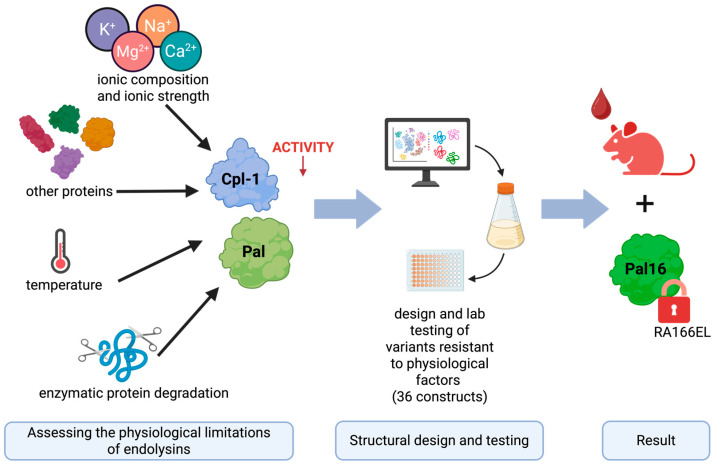
Graphical overview of the engineering approach used to improve the bacteriolytic endolysins Cpl-1 and Pal. The results presented in this manuscript reflect the following steps (from left to right): experimental determination of factors limiting the activity of Cpl-1 and Pal bacteriolytic endolysins, using this knowledge in in silico design variants of Cpl-1 and Pal and testing selected variants in wet-lab experiments. Finally, the best-performing variant was selected for in vivo testing in a mouse model. Created in BioRender. Dąbrowska, K. (2026) https://BioRender.com/h0c0koa (accessed on 29 January 2026).

In this study, we systematically evaluated the major physicochemical and biological factors limiting the activity and stability of Cpl-1 and Pal in blood, integrating published data with experimental findings. We examined the effects of ion concentration, ionic strength, serum total protein concentration, and physiological temperature (compared with room temperature and 50 °C). In addition, we analyzed the amino acid sequences of both enzymes to identify potential blood protease cleavage sites. Based on these analyses, we designed and modeled variants containing targeted amino acid substitutions intended to improve resistance to physiological constraints and enhance antibacterial efficacy in blood-like environments. These variants were subsequently validated experimentally (see Figure 1).

## 2. Materials and Methods

### 2.1. Protein Expression and Purification

The endolysins Pal (acc. no. YP_004306947) (Pal WT, wild-type), derived from *Streptococcus* phage Dp-1, and Cpl-1 (acc. no. CAA87744) (Cpl-1 WT, wild-type), derived from *Streptococcus* phage Cp-1, were used in this study. The genes encoding Pal and Cpl-1 (wild-types, WT), codon-optimized for expression in the *E. coli* B834(DE3) bacterial expression system, were synthesized de novo and cloned into the pBAD_HisA plasmid (BioCat GmbH, Heidelberg, Germany).

Variants of the wild-type endolysins were generated by site-directed mutagenesis of original plasmids using PCR with primers encoding the desired mutations, followed by ligation with Liga5 (A&A Biotechnology, Gdańsk, Poland). All constructs were transformed into *E. coli* B834(DE3) cells (EMD) and cultured at 37 °C with shaking in Luria–Bertani (LB) broth (10 g/L tryptone, 10 g/L NaCl, 5 g/L yeast extract) supplemented with ampicillin (50 mg/L, Sigma-Aldrich, St. Gallen, Switzerland) until the optical density at 600 nm (OD_600_) reached 1.2. Protein expression was induced by adding arabinose to a final concentration of 2.5 g/L (0.25%). Cultures were incubated overnight at 22 °C with vigorous shaking.

Cells were harvested by centrifugation (7000× *g*, 5 min) and resuspended in phosphate-buffered saline (PBS: 140 mM NaCl, 2.68 mM KCl, 1.47 mM KH_2_PO_4_, 6.46 mM Na_2_HPO_4_, pH 7.2) supplemented with phenylmethylsulfonyl fluoride (PMSF, 1 mM) and lysozyme (0.5 mg/mL). The slurry was incubated on ice for 3–7 h on ice and lysed using the freeze–thaw method. Next, Mg^2+^ (up to 0.25 mM), DNase (up to 20 μg/mL), and RNase (up to 40 μg/mL) were added and incubated on ice for an additional 3 h. Cell debris was removed by centrifugation (12,000× *g*, 30 min, 4 °C), and the soluble fraction (supernatant) was collected.

Cpl-1 and Pal (WT and variants) were supplemented with imidazole in PBS up to 75 mM and incubated with NiNTA agarose. The resin was washed with 10 column volumes of 85 mM imidazole in PBS. Bound proteins were eluted with 5 volumes of 250 mM imidazole and dialyzed three times against a 100-fold excess of PBS at 4 °C using a 3 kDa molecular weight cut-off membrane (Spectra/Por, Repligen, Waltham, MA, USA). Purified protein samples were filtered through sterile 0.22 μm polyvinylidene fluoride filters (Millipore, Burlington, MA, USA). Protein purity was monitored by SDS-PAGE at all stages. Protein concentrations were determined using the Bradford assay (Sigma-Aldrich, St. Gallen, Switzerland) according to the manufacturer’s instructions.

### 2.2. Testing Activity, Fluorometric Assay

Sytox™ Green (Thermo Fisher Scientific, Waltham, MA, USA) was used to measure bacteriolytic activity using a fluorescence plate reader, following a slightly modified protocol previously described by the authors [40,43]. Briefly, metabolically active *S. pneumoniae* cells were mixed with the bacteriolytic agent, like endolysin in the presence of Sytox™ Green DNA dye. Upon disruption of the bacterial cell wall, intracellular DNA became accessible to the dye, resulting in increased fluorescence signal emitted by the sample.

*S. pneumoniae* cells in the logarithmic growth phase were cultured in Todd-Hewitt broth supplemented with 2% yeast extract (THY). Cells were harvested by centrifugation (6000× *g*, 5 min, 20 °C), washed twice with PBS by resuspension in the same volume like the original culture, centrifuged under the same conditions, and resuspended in PBS to an OD of 0.3. PBS was selected as a well-characterized buffer commonly used in endolysin studies, ensuring methodological consistency and comparability. It provides a stable pH of 7.4, comparable to physiological conditions, and does not interfere with endolysin activity through carbonate ions.

For the standard assay, 50 µL of bacterial suspension was mixed in a 96-well plate with 50 µL of 5 µM Sytox™ Green solution in PBS (diluted from a 5 mM stock solution in DMSO, ThermoFisher Scientific, to final concentration in the assay: 1.25 µM), 50 µL of the test solution (e.g., murine plasma, ion-containing buffers, or PBS), and finally 50 µL of endolysin solution to initiate the lytic reaction. Typical final endolysin concentrations, unless otherwise stated, were 8 mg/L for Cpl-1 or 4 mg/L for Pal and their respective variants. Total reaction volume was 200 µL.

For control wells lacking bacteria or endolysin, the corresponding volume was replaced with PBS. Immediately after addition of endolysin, fluorescence changes (Excitation/Emission: 504/523 nm) were recorded at room temperature for up to 15 min, with precise time resolution for each measurement. Under standard conditions, bacteriolytic activity of Cpl-1 (8 mg/L) and Pal (4 mg/L) was typically completed within 5 min.

Raw fluorescence data from each well were normalized using a modified Gompertz function, in which the parameter *c* was defined as lytic activity (Equation (1)).

(1) $$f\left(t\right)=ae^{-e^{\left(b^{2}+0.5\right)-ct^{c}}}+y$$

The modified Gompertz equation was fitted to the fluorescence data for each well separately using a least-squares approach, based on fluorescence emission (*f*) and time (*t*) recorded throughout the entire measurement period. Neutral conditions were defined as 100% lytic activity, and the activities of other groups were calculated relative to this control. If the mean and standard deviation (SD) of the *c* parameter in the Gompertz function for a given sample were not significantly different from 0.0 (*p* > 0.05, two-sided simple *t*-test), lytic activity was considered undetectable and recorded as 0% of control. Technical controls in the fluorometric assay were performed as previously described [40].

Detection of lytic activity in murine plasma at final plasma concentrations exceeding 25% required a modified protocol. In these experiments, 10 µL of bacterial suspension in PBS (with pre-added Sytox™ Green, final concentration 1.25 µM) was mixed with 180 µL of murine plasma (diluted with PBS if necessary) and 10 µL of endolysin solution to initiate the reaction. Final endolysin concentrations were 8 mg/L for Cpl-1 and 4 mg/L for Pal.

To assess the effect of ions, the standard assay protocol was modified as follows: 50 µL of bacterial suspension was mixed with 50 µL of 5 µM Sytox™ Green in PBS (from a 5 mM stock in DMSO, final concentration 1.25 µM), 20 µL of PBS, and 30 µL of water or ion solution prepared in water. Finally, 50 µL of endolysin solution was added to initiate lysis. For these experiments (Results Section 3.1), the reference control (100% activity) consisted of 85% PBS diluted with water. Fluorescence measurement settings remained unchanged.

To evaluate the effect of temperature, endolysins were diluted in fresh PBS to four times the final working concentration used in the fluorometric assay and incubated at the indicated temperature. For experiments involving incubation in murine plasma, endolysins prepared in PBS were incubated in 60% murine plasma (in PBS) for 30 or 60 min at concentrations four times higher than those used during measurement. Due to volume constraints of the fluorometric assay, the final plasma concentration during measurement was 15%. Final concentrations of endolysin were the same as standard assay levels (8 mg/L for Cpl-1 and 4 mg/L for Pal).

### 2.3. Bioinformatic Modeling

Modeling of Cpl-1 variants was performed using the Cpl-1 crystal structure available in the Protein Data Bank (PDB accession number: 2IXU). For Pal variants, a structural model previously generated using I-TASSER and described in our earlier publication was used [44].

Alanine scanning was performed using LaserGene software [45,46]. Residues for which substitution with alanine resulted in a substantial increase in ΔΔG (more than 0.5) were considered critical for maintaining tertiary structure and were excluded from further substitutions during variant design. All substitutions were modeled allowing backbone flexibility using the backrub motion algorithm with a cut-off of 0.7 nm.

ConSurf analysis was conducted using the ConSurf server to identify amino acids conserved due to structural stability or kinetic activity [47,48,49,50]. Conserved residues were also excluded from subsequent mutational analysis.

To reduce inhibitory effects of ions, substitutions targeted oppositely charged residues (K, D, E, R) located within 10 amino acids of each other in the primary amino acid sequence and positioned within 0.5 nm in three-dimensional space, particularly on the protein surface and preferably within the cell wall-binding domain (CBD). Such substitutions were designed to eliminate surface dipoles. In most cases, substitution to leucine was energetically favorable, as indicated by minimal folding energy among non-charged amino acids.

To increase thermal stability, salt bridges were created between residues whose Cα atoms were within 1.5 nm in three-dimensional space but separated by more than 10 amino acids in the primary sequence. Buried residues or residues not oriented toward each other were excluded. Selected residues were substituted with complementary charged amino acids (R or K for positive charge and D or E for negative charge) to form stabilizing ionic interactions. Double substitutions that brought oppositely charged side chains into close proximity and significantly reduced folding energy were selected for further experimental testing.

To minimize susceptibility to blood proteases, predicted cleavage sites for proteases expected to be present in blood were eliminated by substituting the relevant residues with chemically similar amino acids that do not form recognized protease cleavage motifs [37,38].

### 2.4. Animal Experiments

For in vivo experiments in a mouse model, endolysin samples were purified to remove lipopolysaccharide (LPS, endotoxin). Endotoxin removal was performed using EndoTrap Blue according to the manufacturer’s instructions (Hyglos GmbH, Munich, Germany). Endotoxin content was measured using the EndoLISA assay (Hyglos GmbH, Munich, Germany) and confirmed to be below 2 endotoxin units (EU)/mL for all samples.

C57BL/6 female mice, same litter (6–10 weeks old, n = 13 per group in two independent experimental replicates) received 200 µL of purified Pal WT, Pal variant 16, or PBS via tail vein injection (0.1 mg protein per mouse). Blood samples were collected at 40, 80, and 160 min post-injection and centrifuged sequentially at 2250× *g* for 5 min and 10,000× *g* for 10 min at 10 °C. Bacteriolytic activity of the collected serum samples was measured using the fluorometric assay as described above, with a reduced final reaction volume of 100 µL.

Briefly, 15 µL of collected serum was mixed with 10 µL PBS, 25 µL of *S. pneumoniae* suspension (logarithmic phase, washed and resuspended twice in PBS to OD 0.3), and 50 µL Sytox™ Green in PBS (final concentration 1.25 µM). No additional endolysin was added and serum from treated mice served as the source of active bacteriolytic agent in the reaction.

### 2.5. Variant Creation

Variants of Cpl-1 and Pal were generated using reverse PCR. Primers were designed such that both forward and reverse primers annealed at the site representing amino acid substitution, with the forward primer encoding the desired mutation at its 5′ end. Plasmids containing the wild-type endolysin genes served as templates.

PCR amplification produced linearized plasmids incorporating the intended mutation. The linear products were ligated using the Liga5 kit (A&A Biotechnology) and transformed into *E. coli* B834 cells. Endolysin variants were subsequently expressed and purified under the same conditions as the wild-type proteins.

## 3. Results

### 3.1. Cpl-1 and Pal Endolysin Activity Is Impaired in High Concentrations of Murine Plasma

**Figure 2:**
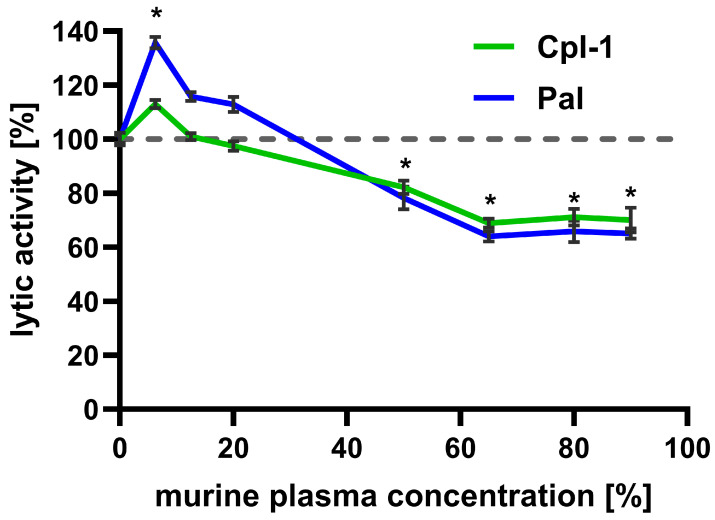
Effect of increasing murine plasma concentration on the bacteriolytic activity of Cpl-1 and Pal endolysins. Data represent Cpl-1 (green) and Pal (blue) bacteriolytic activity in murine plasma as the reaction environment. Plasma concentrations ranged from 0% to 90% (*v*/*v*). Lytic activity in PBS was set to 100% for each endolysin. Lytic activity was measured as the lysis rate of *Streptococcus pneumoniae* cells [40]. Asterisks denote statistically significant differences between lytic activity in PBS (0% plasma) and at the indicated plasma concentration (adjusted *p*-value < 0.05, two-sided Welch’s *t*-test). The dashed grey line represents 100% lytic activity. Points and whiskers represent means and SD of four experimental replicates.

Bacteriolytic activity of Cpl-1 (8 mg/L) and Pal (4 mg/L) was assessed in increasing murine plasma concentrations up to 90% (the highest technically available concentration) and compared to their lytic activity in PBS (Figure 2). Interestingly, low plasma concentrations improved bacteriolytic activity of both enzymes, peaking at 6.5% plasma. This effect was significant for Pal, which achieved almost 140% of its activity in PBS. However, further increases in murine plasma concentration resulted in a constant decrease in lytic activity, observed up to 65% murine plasma (Cpl-1 linear correlation: r = −0.00567, adj. R^2^ = 0.948, *p*-value = 0.0033; Pal linear correlation r = −0.00874, adj. R^2^ = 0.963, *p*-value = 0.00197). At higher concentrations of murine plasma (up to 90%), lytic activity of Cpl-1 and Pal did not demonstrate a further decrease and remained similar, at approximately 70% and 65% of lytic activity in PBS (for Cpl-1 and Pal, respectively, non-zero correlation coefficient *p*-value: 0.592 for Cpl-1 and 0.576 for Pal). The decrease in lytic activity of Cpl-1 and Pal at plasma concentrations of 50% or higher was statistically significant compared to lytic activity in PBS.

### 3.2. Magnesium and Calcium Ions Present in Blood Have an Inhibitory Effect on Lytic Activity of Endolysins

**Figure 3:**
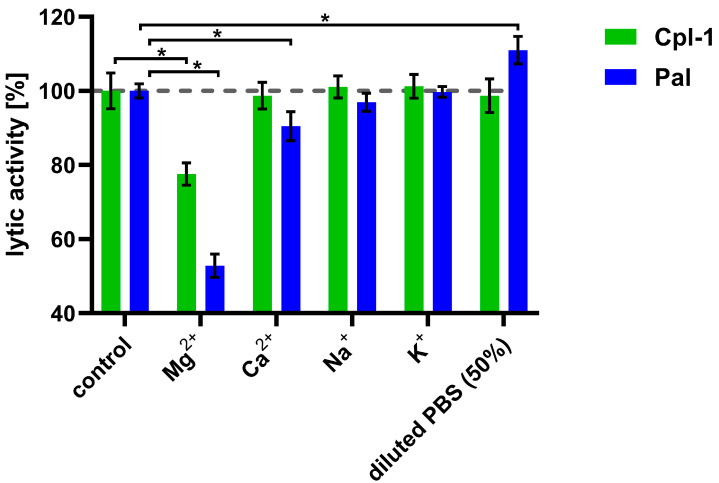
Effects of various ions on the bacteriolytic activity of Cpl-1 and Pal. Bars represent lytic activity of Cpl-1 (green) and Pal (blue) endolysins in the reaction environment (127.5 mM Na^+^, 3.53 mM K^+^, 119 mM Cl^−^, 6.74 mM HPO_4_^2−^) or in PBS supplemented with ions (0.225 mM Mg^2+^, 0.375 mM Ca^2+^, 149 mM Na^+^, and 4.28 mM K^+^). Lytic activity was measured as the lysis rate of *Streptococcus pneumoniae* cells [40]. Lytic activity under control conditions was set to 100%. Asterisks denote *p*-values < 0.05 (two-sided Welch’s *t*-test). The dashed grey line represents 100% lytic activity. Bars and whiskers represent means and SD of three experimental replicates.

After observing the detrimental effect of plasma on the bacteriolytic activity of the Cpl-1 and Pal endolysins, we aimed to identify the underlying causes by examining the impact of divalent ions, ionic strength, and plasma proteins on their activity. To determine whether, and to what extent, major ions present in blood are involved in the decrease in endolysin activity, we determined the impact of sodium, potassium, calcium, and magnesium ions on Cpl-1 and Pal activity at concentrations similar to those present in blood (Figure 3) [36,37,51]. Magnesium ions at a concentration of 0.225 mM had a significant detrimental effect on the activity of both endolysins: 22% for Cpl-1 and 47% for Pal (*p* < 0.05, two-sided Welch’s *t*-test). Calcium ions at a concentration of 0.375 mM reduced bacteriolytic activity of Pal by 10% (*p* < 0.05, two-sided Welch’s *t*-test) but did not alter activity of Cpl-1. Sodium (149 mM) and potassium (4.28 mM) ions had no significant impact on lytic activity. Manipulating ionic strength, in the absence of ions that directly affect endolysin activity, can also be considered to improve lytic activity of endolysins. Interestingly, lower ionic strength (decreased from 133, as in PBS, to 78.5) improved lytic activity of Pal (*p* < 0.05, two-side Welsh’s *t*-test, Figure 3) (see ion concentrations in Supplementary Table S1).

### 3.3. Combination of Ions Under Physiological Conditions Has Negative Effect on Bacteriolytic Activity of Endolysins

Next, we assessed the impact of combined major divalent ions (Ca^2+^ and Mg^2+^) on endolysin activity, using concentrations corresponding to physiological levels in blood (2.5 mM Ca^2+^ and 1.5 mM Mg^2+^), as well as concentrations twice as high [52,53].

**Figure 4:**
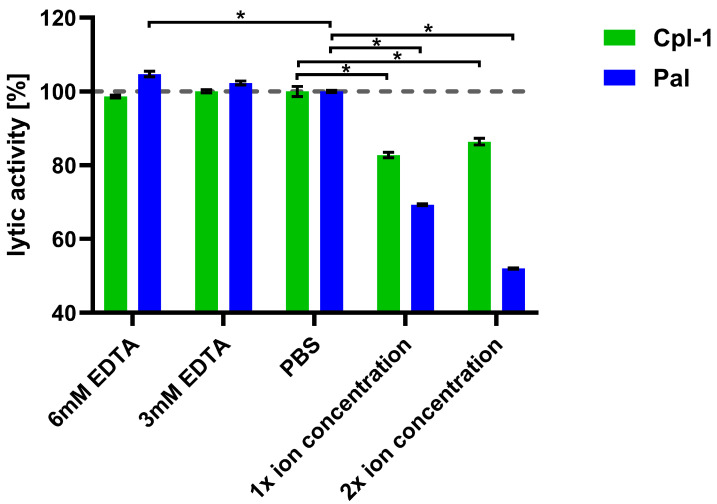
Joint effects of Ca^2+^ and Mg^2+^ ions on lytic activity of Cpl-1 and Pal. Bars represent mean lytic activity of Cpl-1 (green) and Pal (blue) endolysins in EDTA, PBS (no Mg^2+^ or Ca^2+^), 1× ion concentration (2.5 mM Ca^2+^ and 1.5 mM Mg^2+^ in PBS, as expected in blood), and 2× ion concentration (5.0 mM Ca^2+^ and 3.0 mM Mg^2+^ in PBS). Lytic activity was normalized to activity in PBS (set to 100%). Lytic activity was measured as the lysis rate of *Streptococcus pneumoniae* cells caused by the respective endolysin [40]. Bars and whiskers show means and SD from three experimental replicates. Asterisks denote *p*-values < 0.05 (two-sided Welch’s *t*-test). The dashed grey line represents 100% lytic activity.

The lytic activity of Cpl-1 and Pal was measured in the presence of both ions at these defined levels. To control for potential interference from ions released by bacterial cells, parallel assays with EDTA were included (Figure 4). Bacteriolytic activity in PBS was used as a reference (100%). Lytic activity of Pal dropped by 31% (*p* < 0.05, two-sided Welch’s *t*-test) in the presence of 2.5 mM Ca^2+^ and 1.5 mM Mg^2+^ and dropped further at higher concentrations of the tested ions, showing marked impairment of endolysin activity by these ions. This antagonistic effect of Ca^2+^ and Mg^2+^ suggests their shielding activity. A further decline of Pal activity was observed at increased ion concentrations (5.0 mM Ca^2+^ and 3.0 mM Mg^2+^) (Figure 4). Pal also showed a slightly increased (5%) activity in 6 mM EDTA, suggesting that even ions released from bacteria had a detectable inhibitory effect on Pal (*p* < 0.05, two-sided Welch’s *t*-test). Cpl-1 showed reduced activity in the presence of the tested ions by 17%. However, in contrast to Pal, no increased activity in 3 or 6 mM EDTA was detected. This demonstrates that endolysins differ in their susceptibility to ion-related effects on bacteriolytic activity. Importantly, removal of divalent ions by EDTA did not lower activity of Cpl-1 or Pal, suggesting that such ions are not required or crucial as cofactors for bacteriolytic activity of these enzymes.

### 3.4. The Concentration of Bystander Proteins Has an Inhibitory Effect on Pal Endolysin

**Figure 5:**
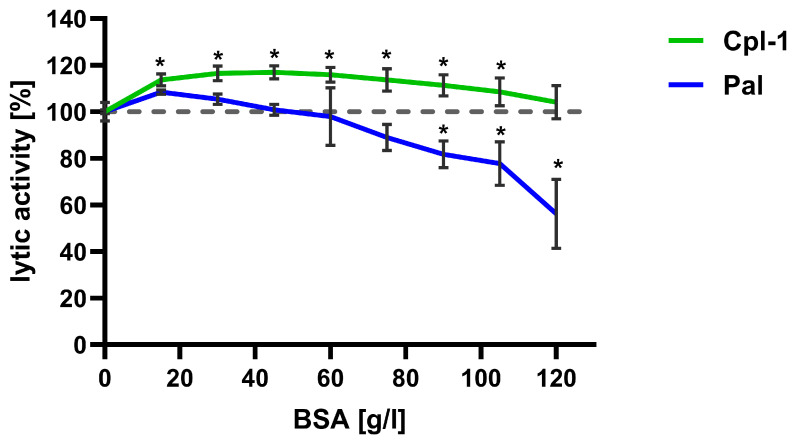
Effect of bystander proteins on the bacteriolytic activity of Cpl-1 and Pal endolysins. Lytic activity of Cpl-1 (green) and Pal (blue) was measured at various concentrations of BSA as a non-interacting bystander protein. Lytic activity was normalized to activity in PBS without BSA (set to 100%). Lytic activity was measured as the lysis rate of *Streptococcus pneumoniae* cells caused by the respective endolysin [40]. Points and whiskers show means and SD from three experimental replicates. Asterisks denote statistically significant differences between lytic activity at the indicated BSA concentration and in PBS alone (*p* < 0.05, two-sided Welch’s *t*-test). The dashed grey line represents 100% lytic activity.

The lytic activity of Cpl-1 and Pal was further evaluated at increasing concentrations of bovine serum albumin (BSA), used as a non-interacting bystander protein, to assess the potential impact of high protein levels in blood on endolysin activity. Lytic activity was tested in PBS with BSA concentrations up to 120 g/L (Figure 5). Notably, the expected concentration of proteins in blood is lower than 85 g/L. Endolysin activity in PBS without BSA was used as a reference and set to 100%.

For Cpl-1, an increase in activity of approximately 10% was observed at all tested protein concentrations compared to PBS; difference was statistically significant up to a BSA concentration of 100 g/L (*p* < 0.05, two-sided Welch’s *t*-test). Conversely, Pal lytic activity increased insignificantly at the lowest BSA concentrations, and from approximately 40 g/L, its activity decreased with increasing BSA concentration. Activity reached a statistically significant reduction of 10% at 75 g/L BSA and then diminished further (Figure 5).

### 3.5. pH in Blood Is in Optimal Range for Cpl-1 and Pal Activity

Lytic activity of Cpl-1 and Pal was measured at different pH levels to assess whether pH may limit their activity in blood. We observed no significant change in activity for Cpl-1 or Pal within the pH range from 6.5 to 8.5, suggesting that blood pH provides optimal conditions for their enzymatic reaction (Supplementary Table S5).

### 3.6. Body Temperature Has Inhibitory Effect on Cpl-1 and Pal Activity

**Figure 6:**
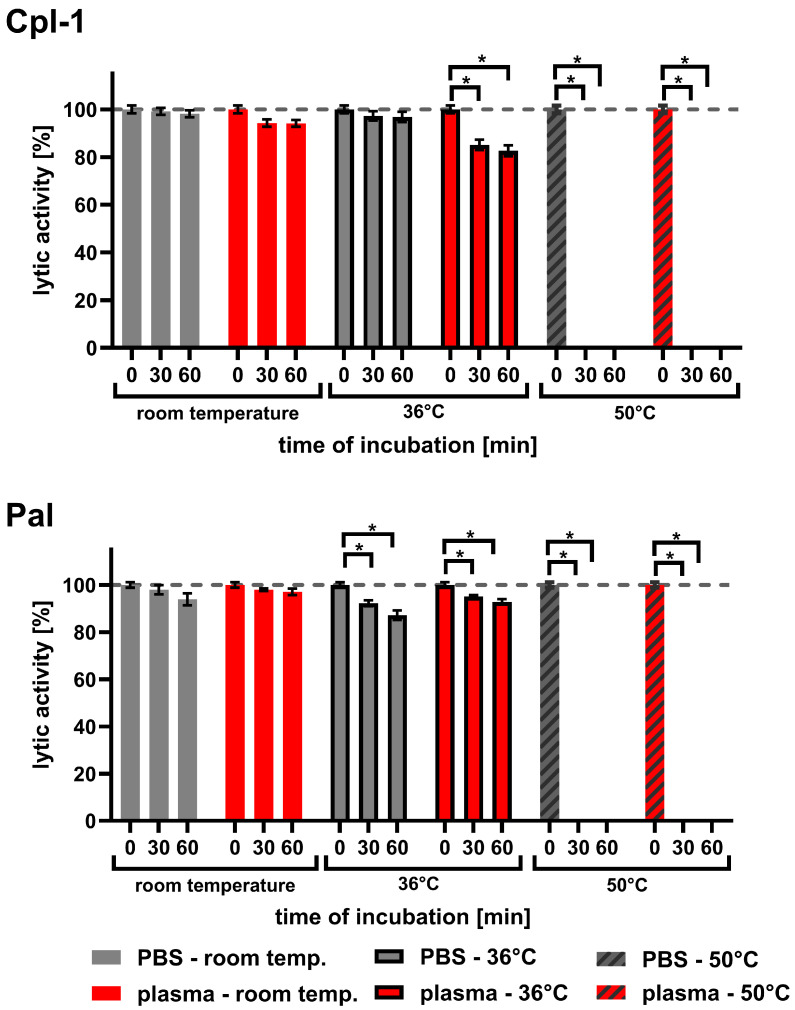
Lytic activity of Cpl-1 and Pal after incubation in PBS or murine plasma at varying temperatures. Lytic activity of Cpl-1 and Pal was assessed after incubation in either PBS (grey) or 60% murine plasma (red) at room temperature (RT), 36 °C, or 50 °C for 30 or 60 min. Activity was measured as the lysis rate of *Streptococcus pneumoniae* cells [40]. For each condition, baseline (unincubated, 0 min) activity of the respective endolysin was set to 100%. Plasma was used at the highest concentration technically achievable (60%). Bars and whiskers represent means and SD of six experimental replicates. Asterisks denote *p*-values < 0.05 (two-sided Welch’s *t*-test). The dashed grey line represents 100% lytic activity.

Since endolysins used in medical applications must be exposed to body temperature for longer periods than during typical laboratory testing, we investigated the effect of temperature on Cpl-1 and Pal. The experiments included incubations in both PBS and plasma for up to 60 min at 36 °C, compared to incubations at room temperature (RT) and 50 °C (Figure 6 and Table 1). Sixty minutes of incubating Cpl-1 and Pal at 36 °C in plasma resulted in 17.3% and 7.2% activity loss, respectively (*p* < 0.05, two-sided Welch’s *t*-test, three experimental replicates). Incubation of Cpl-1 and Pal at 50 °C for 30 or 60 min caused complete loss of lytic activity in PBS and in plasma. We did not observe a significant loss of lytic activity after incubating Cpl-1 at 36 °C in PBS for 30 or 60 min; however, we observed loss in plasma, suggesting that the blood environment can increase sensitivity to body temperature in some endolysins.

### 3.7. Creation of In Silico Variants

We used observations of endolysin activity under different conditions to design variants of Cpl-1 and Pal that were less prone to the inhibitory factors identified in the experiments. Variants were created by amino acid substitutions in Cpl-1 and Pal. First, by alanine scanning, we identified amino acids crucial for structural stability. We applied a previously published Cpl-1 model (PDB: 2IXU) and Pal models [44,54]. If substitution of an amino acid with alanine increased the folding energy of the protein (ΔΔG) significantly, this amino acid was marked as crucial for the tertiary structure, and such amino acids were exempted from substitutions (Supplementary Tables S3 and S4). We also analyzed Pal and Cpl-1 with ConSurf software to identify amino acids conserved due to structural stability, substrate binding, or kinetic activity [47,48,49,50]. Such amino acids were also exempted from substitutions (Supplementary Figures S1 and S2). This stage of analysis also allowed us to identify amino acids exposed on the protein surfaces.

We hypothesize that shielding effects are the primary cause of the inhibitory impact observed at elevated Mg^2+^ and Ca^2+^ concentrations, which reduced lytic activity of the endolysins (Figure 3 and Figure 4). This effect was stronger for Pal. Moreover, EDTA slightly increased its lytic activity, indicating that even ions released from destroyed bacterial cells have a measurable inhibitory effect on Pal. The EDTA effect was not observed for Cpl-1, and Mg^2+^ inhibited this endolysin to a lesser extent than Pal. Mg^2+^ concentrations comparable to those in blood strongly inhibited the lytic activity of Cpl-1 and Pal. However, Ca^2+^ ions showed a markedly weaker inhibitory effect despite being present at higher concentrations than Mg^2+^. Some previous reports indicate a stabilizing effect of Ca^2+^ and Mg^2+^ ions on the bacterial cell-wall, lowering lytic activity of endolysins [33].

We used the aforementioned observations to design variants of endolysins with enhanced efficacy under physiological ionic conditions, and we modified electrostatic interactions without changing the overall charge of the protein. This was achieved by removing dipoles from the protein surface. Specifically, we reduced the number of oppositely charged amino acids located close to each other on the surface, preferably in the cell-wall binding domain. When such residues are in close proximity, they can form surface dipoles. Removing these residue pairs does not change the overall charge of the protein; however, surface dipoles can affect how the protein interacts with ions in the surrounding solution. Fewer dipoles, even without altering total charge, reduce the number of ions gathering around the protein, especially near the cell-wall binding domain. We considered the modular structure of endolysins, avoided altering known catalytic or substrate-binding residues, and focused instead on testing changes to surface charges within the CBD to modulate CBD-substrate interactions by adjusting electrostatic forces that drive binding. This is expected to limit ion interference during interactions between the endolysin CBD and the bacterial cell-wall. In practice, we removed oppositely charged surface amino acids (K, D, E, R) that were close in the amino acid sequence (less than 10 residues apart) and whose charged atoms were less than 0.5 nm from each other. We prioritized residues located near the suspected binding site between the CBD and the bacterial cell-wall, selecting pairs whose removal caused the smallest increase in ΔΔG.

To increase thermal resistance, we created salt bridges. We selected non-charged amino acid pairs with Cα atoms located close to each other in 3D space (less than 1.5 nm apart), but more than 10 amino acids apart in the amino acid sequence. These residues were located on the protein surface and oriented toward each other. Such pairs were considered likely to form salt bridges after substitution with oppositely charged amino acids. We then substituted them with charged amino acids to create a pair of positively charged (R or K) and negatively charged (D or E) residues. Double substitutions that brought the charged side chains close together and strongly lowered ΔΔG were selected for further testing. These engineered salt bridges were expected to enhance structural stability and improve thermal resistance without disrupting protein function.

At this stage, we also implemented protection of endolysins from blood proteases by removing known protease cleavage sites, targeting those expected to be present in blood [37,38]. We used the most chemically similar amino acids to introduce substitutions that disrupted cleavage sites (Table 1).

Although blood pH falls within the optimal range for the investigated endolysins (Supplementary Table S5), we included Cpl-1 and Pal variants with potentially increased resistance to pH levels outside their optimal range during exposure in blood. This may help extend the application of these enzymes to environments such as urine or to challenging physiological conditions such as sepsis, which can involve atypical disturbances in blood pH. This was addressed by substituting histidine with tyrosine to test whether this change could stabilize the endolysins and increase their longevity in the bloodstream.

In summary, we selected 36 variants of Cpl-1 and Pal endolysins for laboratory validation (Table 1).

### 3.8. Validation of Cpl-a and Pal Variants Activity

**Figure 7:**
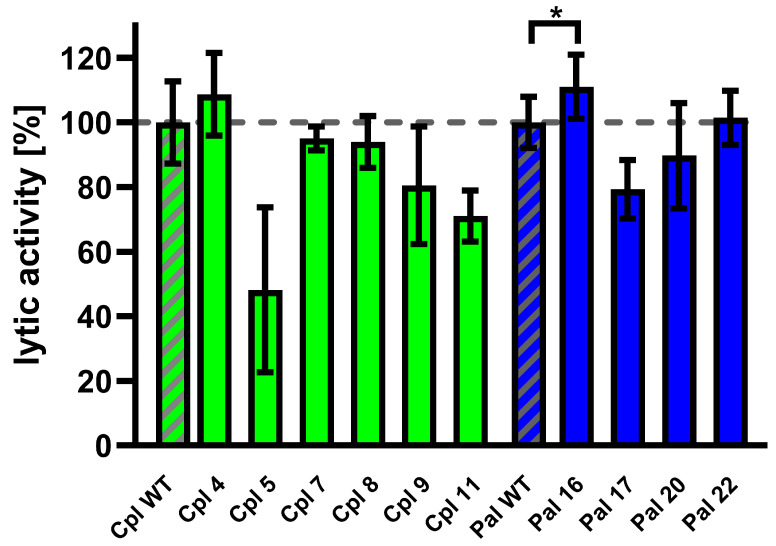
Activity of Cpl-1 and Pal variants in plasma. Lytic activity of Cpl-1 variants (green) and Pal variants (blue) was measured ex vivo in 15% murine plasma in PBS as the lysis rate of metabolically active *Streptococcus pneumoniae* cells caused by the respective endolysin [40]. Lytic activity of the wild-type endolysin was set to 100% and is marked with additional diagonal grey stripes. Bars and whiskers represent means and SD of three replicates. Asterisks denote *p*-values < 0.05 (one-sided *t*-test). The dashed grey line represents 100% lytic activity.

Variants of Cpl-1 and Pal were created by reverse PCR or double reverse PCR using synthesized primers containing the designed modifications. Plasmids encoding the amino acid sequence of the wild-type endolysins were used as templates. Constructed expression vectors were used in an *E. coli* expression system, and 10 variants yielded sufficient protein production for further testing: Cpl4, Cpl5, Cpl7, Cpl8, Cpl9, Cpl11, Pal16, Pal17, Pal20, and Pal22. Tested endolysins (variants and wild type) were purified, and their activity was evaluated in murine plasma at 36 °C. One variant (Pal16) showed significantly higher activity than the wild type (Figure 7 and Supplementary Table S2).

Pal16 was further tested in vivo in C57BL/6 mice. Animals were inoculated with 0.1 mg/mouse of the Pal16 variant, wild-type Pal, or PBS via injection into the lateral tail vein. Blood was collected after 40, 80, and 160 min and added to *Streptococcus pneumoniae* culture and bacteriolytic activity of endolysins present in blood was detected using the Sytox Green assay [40]. Blood collected from mice treated with Pal16 showed 15% higher activity than blood from mice treated with wild-type Pal after 160 min (*p* = 0.037, one-sided *t*-test). No antibacterial activity was observed in blood collected from control animals treated with PBS.

## 4. Discussion

This study aimed to identify physiological factors in the human body that may limit the use of endolysins as potential biological drugs against bacterial infections. We focused on experimentally assessing the inhibitory effects of physiological factors such as ions, bystander proteins, temperature, pH, and proteases on endolysins. Overall assessment demonstrated that the activity of Cpl-1 and Pal was inhibited by murine plasma, with inhibition reaching approximately 30%. As plasma concentration increased from 20% to 60%, endolysin activity decreased in an inversely proportional manner. However, at higher plasma concentrations, the inhibitory effect remained at around 30%, suggesting a possible saturation effect. This may reflect either (1) the impact of physical properties of the reaction environment on enzyme activity, such as ionic strength, or (2) a probable non-competitive inhibitory effect of plasma, e.g., altered energy of interaction between the cell-wall binding domain and the bacterial cell wall in the presence of calcium and magnesium ions. Notably, the slight initial increase in activity at low plasma concentrations highlights the complexity and heterogeneity of plasma as a biological medium. Its effect on protein activity is not merely the sum of individual physicochemical and biological factors, but rather the result of multiple synergistic and antagonistic interactions occurring simultaneously.

Unexpectedly, we observed enhanced endolysin activity at low plasma concentrations. Notably, the increase in endolysin activity in diluted serum was, on average, consistent with the effects exerted by bystander proteins such as albumin (Figure 5), which also enhanced activity at concentrations lower than those present in blood. At the same time, the investigated endolysins proved to be insensitive to ionic dilution (Figure 4). Taken together, these findings suggest that diluted serum may provide near-optimal conditions for bacteriolytic activity of endolysins. This observation further underscores the heterogeneous nature of plasma, which acts as a dynamic environment generating multiple synergistic and antagonistic interactions that occur simultaneously and continuously counterbalance one another. Nevertheless, the dominant effect of the blood environment on Cpl-1 and Pal was clearly inhibitory, with a significant loss of antibacterial potency.

To explore what might be responsible for inhibiting endolysins in plasma, we examined ion levels mirroring those found in blood. Under these conditions, we detected a clear reduction in endolysin activity (Figure 2 and Figure 3). Magnesium ions, in particular, markedly decreased activity of both Cpl-1 and Pal, and this inhibitory effect occurred at concentrations typical of mammalian blood. These observations are consistent with the current understanding of endolysin function and with proposed mechanistic models suggesting that divalent cations influence stability of the bacterial cell wall, making it more resistant to endolysin-mediated degradation [22,32,33,55]. In addition, when proteins present charged amino acids on their surfaces, they attract polar solvent molecules and dissolved ions (such as Mg^2+^ or Ca^2+^). These ions accumulate around the proteins and “shield” them from other proteins or molecules, including enzyme substrates such as bacterial cell-wall components. Because lytic activity of Pal increased after addition of EDTA to the baseline reaction (reaction buffer did not contain Mg^2+^ or Ca^2+^), divalent ions likely originated from lysed bacterial cells. This suggests that endolysins can be sensitive even to trace amounts of these ions, which may play a critical role in modulating endolysin function and catalytic activity. Because endolysin-specific profiles of inhibitory ionic conditions were observed, for instance calcium ions affected Pal but had a lesser impact on Cpl-1, our observations warrant further investigation into ion-endolysin interaction mechanisms.

We also evaluated whether prolonged exposure to body temperature affects activity. After 60 min of incubation at physiological temperature (36 °C), both endolysins showed a marked reduction in activity. Although the in vivo circulation time of endolysins used as therapeutic antibacterials is not yet well defined, our findings clearly indicate that these enzymes are sensitive to elevated temperature. This suggests that thermal resistance in blood-like environments should be an important aspect of preclinical adaptation of endolysins for therapeutic applications.

Endolysins, typically tested in laboratory cultures using standard media, must function in vivo in environments rich in mammalian proteins. These proteins, though non-interacting and acting as bystanders, can change conditions and affect enzymatic reactions in a non-specific manner. Specific interactions between Cpl-1/Pal and serum albumin remain unknown. BSA is a widely used protein in experimental procedures, often serving as a blocking agent or control, particularly in immunoassays. In our previous studies, BSA was also employed in immunoassays evaluating Cpl-1 and Pal. Because those experiments showed normal efficacy, the occurrence of strong specific interactions seems improbable [44]. However, we acknowledge that certain interactions between bacteria and serum can have significant implications for the development of novel antibacterial treatments. Many bacteria, e.g., *S. aureus*, expresses a wide range of proteins that bind host proteins, including serum albumin [56,57,58]. Some albumin-binding proteins, such as Ebh, contribute to bacterial survival in the bloodstream and play a role in the overall pathogenesis of bacterial infections [57]. Also, some studies have explored stabilizing effects of serum albumin on protein therapeutics. For instance, fusing an endolysin with an albumin-binding domain was reported to increase protein half-life in serum [13].

In summary, we assessed the impact of bystander proteins using BSA. Interestingly, Cpl-1 showed elevated activity at BSA concentrations from 20 to 100 g/L (Figure 5). Pal, however, demonstrated significant inhibition of its activity at BSA concentrations of 70 to 120 g/L and no significant effects at lower concentrations (Figure 5). Thus, physiological protein concentrations do not appear to be limiting factor for Cpl-1 when applied in vivo, whereas for Pal they may potentially limit antibacterial activity.

Among the major limitations of this study, one should consider the ion selection, which included macroelements, while specific effects of microelements cannot be excluded. In addition, our experimental testing and the resulting bioinformatic design were based on major assumptions and did not include cellular responses to endolysins that may play a significant role in vivo. For instance, foreign proteins can be captured and neutralized by macrophages and other immune cells. Previous research shows that endolysins can penetrate eukaryotic cells [59], indicating a direction for future studies aimed at bringing endolysins closer to successful medical application. Additional biochemical, biophysical, and proteolytic characterization of the endolysin mutants is also an important direction for future work, as it could help clarify how these variants function in body-like environments.

Another limitation concerns proteolytic inactivation of Cpl-1 and Pal and should be discussed further. There are many mechanisms by which therapeutic proteins can be removed or inactivated after delivery to a patient. Proteolytic degradation, renal and hepatic elimination, and specific inactivation (e.g., by antibodies) are among the most commonly discussed [60]. Specific recognition and inactivation were addressed earlier in work published by our group [44]. Renal elimination primarily affects small proteins (below 30 kDa), whereas hepatic inactivation, like specific inactivation, often requires recognition of specific domains [60]. Considering existing evidence, we focused on proteolytic inactivation as the most likely contributor to the loss of endolysin activity detected in blood. However, other mechanisms mentioned here (and others not discussed) cannot be excluded, and further research is needed to determine their roles and importance in inactivation of proteins in blood.

A common way to evaluate enzyme activity and compare it with modified variants is to calculate *k_cat_* and *K_m_* parameters according to the Michaelis-Menten equation. However, critical issues arise that do not occur in analysis of typical enzymes, making such analysis unsuitable and uninformative in case of endolysins. These issues originate from the fundamentally different mechanism of endolysin action. Rather than forming a typical enzyme-substrate complex that transforms into an enzyme-product complex, a crucial first step for endolysins is a formation of interactions between the cell-wall binding domain (CBD) and bacterial cell-wall elements, such as carbohydrates or choline-containing molecules [61,62]. Then, the enzymatically active domain (EAD), in close cooperation with the CBD, is directed toward peptidoglycan (PG) to form a more typical enzyme-substrate complex. CBDs are extremely diverse and may require specific structural features of the bacterial cell-wall surface, thereby impacting the EAD and consequently altering its kinetic activity [63,64]. Such interactions are often a focus in discussions of strain-specificity of endolysins and their varying activity even toward closely related bacterial strains [20,65]. The CBD and EAD are also strongly interconnected intramolecularly. Conformational changes in the CBD can affect EAD activity, sometimes rendering endolysins inactive under certain environmental conditions [66,67,68]. This close cooperation effectively couples resistance to environmental inhibition (“armour”) with catalytic efficiency of EAD (‘engine’) into one. A change in a potential proteolytic site within the CBD may therefore influence the EAD, while simultaneously affecting CBD interactions with the bacterial cell-wall and proteolytic resistance.

Consequently, *k_cat_* and *K_m_* values are reported only rarely for endolysins. When such values are reported, they are often presented as apparent parameters (e.g., “*k_cat_*/*K_m_* apparent”), derived from indirect and assay-dependent measurements, which makes interpretation difficult and limits their value [69]. The established mechanism of bacterial cell lysis introduces additional issues crucial in understanding problems with measurements of endolysin activity. To lyse the bacterial cell wall, an endolysin must create a hole or groove large enough for the cell to burst, a process that is difficult to quantify. This has led some researchers to treat endolysins as antimicrobials and characterize them using minimum inhibitory concentrations (MIC) or related measures [65,70]. Others have proposed evaluating lytic activity using sigmoidal models such as the one used in this manuscript.

Another approach to estimating *k_cat_* and *K_m_* could be use of purified, homologous substrate, but additional problems limit its usefulness. First, purified PG is a polymeric network cross-linked in various ways, often with chemical modifications that depend strongly on the bacterial strain and the purification method. PG is heterogeneous, poorly soluble in water, and variable in composition, all of which strongly influences measured activity of EADs. These issues have contributed to a long-standing discussion of what “purified PG” means when discussing endolysin activity.

A sigmoidal function, such as the modified Gompertz function presented in this manuscript, is more likely to capture endolysin kinetics under these conditions. The parameter *c* in the Gompertz function is defined here as endolysin activity, as it determines the rate of signal increase. The parameter *b* describes a delay between addition of the endolysin and onset of signal increase. Parameters *a* and *y* are linked to technical aspects, with *y* parameter describing the signal level at the beginning of the experiment, while *a* reflects the change between the start and end of the experiment, interpreted as lysis of all bacteria in the sample. Both *a* and *y* depend on the amount of Sytox Green dye and the measured background. The difference between Pal16 and Pal WT was observed in parameter *c*, which was used as the measure of lytic activity (see Figure 7). No significant difference in parameter *b* between Pal16 and Pal WT was detected.

We applied observations from the experimental part of this study to engineer Cpl-1 and Pal variants with improved efficacy under physiological conditions (Table 1). However, most variants did not meet the requirements for efficient production or baseline activity. Only 10 variants were successfully produced in the bacterial expression system, while the remaining variants yielded insufficient amounts of protein. Of the 10 tested variants, only Pal16 demonstrated improved performance in blood circulation, as shown in the mouse model (Figure 7). In this variant, a potential proteolytic cleavage site was modified by changing arginine and alanine at positions 166 and 167 to glutamic acid and leucine (RA166EL). This disrupted a sequence previously reported as a thrombin target (P/A/V/L)R|(S/A) or as a target for other, not fully defined blood proteases (R/K/N)|(S/A/G/V) [71,72]. Notably, the proposed mechanism, which relies on disruption of a thrombin-like cleavage site, has yet to be fully verified. In future studies, the contribution of proteolytic cleavage could be investigated using protease digestion profiling, mass spectrometry mapping of cleavage sites, or specific inhibitor controls.

The other modified variants did not show improved activity in the mouse model; most exhibited reduced activity. It is likely that the substantial loss of activity observed in many variants reflects unintended disruption of CBD-substrate interactions. We chose to include these negative results because we believe they may be useful for other researchers working on endolysin protein engineering.

These findings underscore the complexity of engineering endolysins for use in mammalian systems. Achieving resistance to physiological stressors such as elevated temperature, ionic strength, or pH deviations remains a significant challenge and a subject for future investigation. Nevertheless, our results highlight enzyme-specific variability in sensitivity to these factors, which may guide future design strategies. While most of the tested modifications intended to improve physicochemical resilience were unsuccessful, we successfully enhanced circulation stability by removing a blood protease cleavage site, demonstrating that even targeted single-site changes can yield functional improvements.

In summary, our work highlights both the potential and the limitations of engineered endolysins in complex biological environments. These findings may inform future design efforts aimed at optimizing antibacterial enzymes for therapeutic use.

## Figures and Tables

**Table 1:** Cpl-1 and Pal variants designed in silico based on previous experimental observations. The table lists all Cpl-1 and Pal endolysin variants selected after in silico analysis for laboratory verification. The “variant number” column lists the endolysin name with the number later used for variant identification. “Position of modification” indicates amino acid positions in the protein sequence that were substituted. “Original amino acid” and “modified amino acid” list residues in the wild-type endolysin and their substitutions in the variant, respectively. The “code” column describes the substitution code. Many variants contain double substitutions, shown as one variant name linked to two rows.

Protein	Variant Number	Position of Modification	Original Amino Acid	Modified Amino Acid	Code
Cpl-1	Cpl 1	6	D	R	D6R
		276	D	E	D276E
	Cpl 2	269	S	R	S269R
		318	T	E	T318E
	Cpl 3	242	S	R	S242R
		268	G	E	G268E
	Cpl 4	207	KG	EL	KG207EL
	Cpl 5	236	KG	EL	KG236EL
	Cpl 6	298	RG	EL	RG298EL
	Cpl 7	308	KS	EL	KS308EL
	Cpl 8	311	KG	EL	KG311EL
	Cpl 7	246	K	L	K246L
		249	E	L	E249L
	Cpl 8	205	D	L	D205L
		207	K	L	K207L
	Cpl 9	255	K	L	K255L
		256	D	L	D256L
	Cpl 10	311	K	L	K311L
		318	N	L	N324L
	Cpl 11	330	K	L	K330L
		333	D	L	D333L
	Cpl 12	247	D	L	D247L
		250	K	L	K250L
	Cpl 13	203	K	L	K203L
		243	E	L	E243L
	Cpl 14	194	D	L	D194L
		212	R	L	R194L
	Cpl 15	225	K	L	K225L
		334	D	L	D334L
	Cpl 16	182	D	L	D182L
		183	K	L	K182L
	Cpl 17	14	H	Y	H14Y
	Cpl 18	60	H	K	H60K
	Cpl 19	132	H	K	H132K
Pal	Pal 10	252	E	A	E252A
		263	I	R	I263R
	Pal 11	7	KG	EL	KG7EL
	Pal 12	16	KG	EL	KG16EL
	Pal 13	90	KG	EL	KG90EL
	Pal 14	43	RS	EL	RS43EL
	Pal 15	81	RS	EL	RG81EL
	Pal 16	166	RA	EL	RA166EL
	Pal 17	183	KS	EL	KS183EL
	Pal 18	258	KS	EL	KS258EL
	Pal 20	281	K	L	K281L
		251	D	L	D251L
	Pal 21	124	H	Y	H124Y
	Pal 22	202	H	Y	H202Y
	Pal 23	111	H	Y	H111Y
	Pal 24	90	H	Y	H90Y
	Pal 25	60	H	Y	H60Y

## Data Availability

The original contributions presented in this study are included in the article/Supplementary Materials. Further inquiries can be directed to the corresponding authors.

## Supplementary Materials

The following supporting information can be downloaded at: https://www.mdpi.com/article/10.3390/cimb48020231/s1.
