# Supplementary material for: Physicochemical and Proteolytic Barriers Limiting Activity of Cpl-1 and Pal Endolysins in Human Circulation
Source: Curr Issues Mol Biol. 2026 Feb 21;48(2):231. doi: 10.3390/cimb48020231 (PMC12938970; doi:10.3390/cimb48020231)
Supplement: Supplementary file 1 [file cimb-48-00231-s001.zip › SuppMaterials.v2/Supplementary Figure S2.pdf]

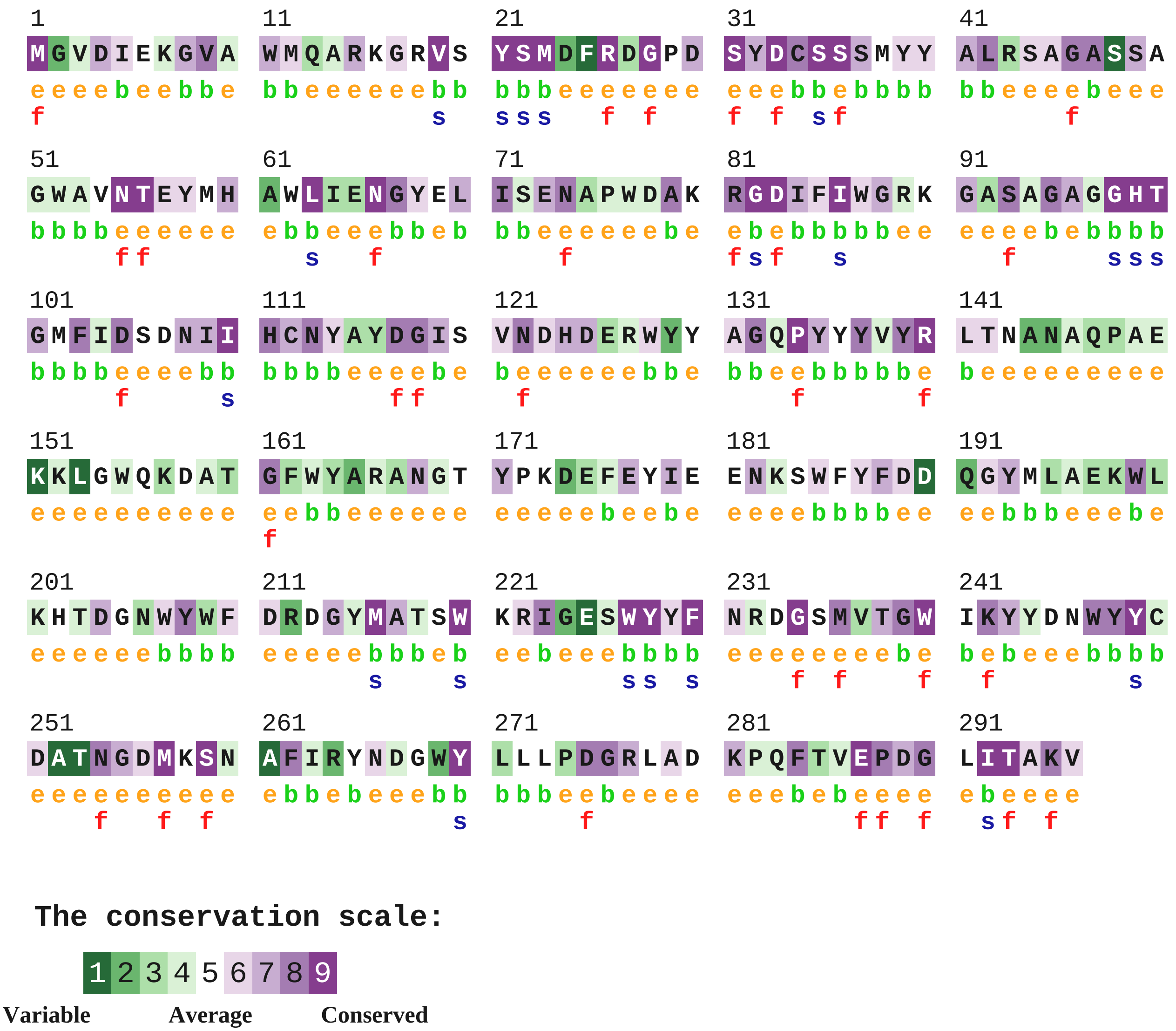

e - An exposed residue according to the neural network algorithm.

b - A buried residue according to the neural network algorithm.

f - A predicted functional residue (highly conserved and exposed).

s - A predicted structural residue (highly conserved and buried).

x - Insufficient data - the calculation for this site was performed on less than 10% of the sequences.
